# Supplementary material for: Development of chemokine network inhibitors using combinatorial saturation mutagenesis
Source: Commun Biol. 2025 Apr 3;8:549. doi: 10.1038/s42003-025-07778-6 (PMC11969024; doi:10.1038/s42003-025-07778-6)
Supplement: Supplementary file 4 — Supplementary Data 2 [file 42003_2025_7778_MOESM4_ESM.zip › SupplementaryData3/scripts/xgeo.tar!/./script/analysis/JK/JK_Human atherosclerosis plaque_SuppTableChemokinePoolConstruction.docx]

Supplementary Table: Human atherosclerosis plaque Chemokine Pool Construction

| Chemokine | Supplier | Code | Concentration [uM] |
| --- | --- | --- | --- |
| CCL2 | Peprotech | 300-04 | 2.6107 |
| CCL18 | Peprotech | 300-34 | 1.85075 |
| CXCL12 | Peprotech | 300-28A | 1.23199 |
| CCL3 | Peprotech | 300-08 | 0.62004 |
| CXCL16 | Peprotech | 300-55 | 0.5649 |
| CCL4 | Peprotech | 300-09 | 0.43035 |
| CXCL8 | Peprotech | 200-08 | 0.34388 |
| CCL14 | Peprotech | 300-38B | 0.32441 |
| CCL8 | Peprotech | 300-15 | 0.27733 |
| CCL4L1 | Peprotech | 300-58 | 0.21912 |
| CCL5 | Peprotech | 300-06 | 0.20515 |
| CXCL7 | Peprotech | 300-14 | 0.18593 |
| CCL19 | Peprotech | 300-29B | 0.17573 |
| CXCL10 | Peprotech | 300-12 | 0.15205 |
| CCL13 | Peprotech | 300-24 | 0.12873 |
| CXCL2 | Peprotech | 300-39 | 0.10247 |
| CCL21 | Peprotech | 300-35A | 0.08433 |
| CXCL9 | Peprotech | 300-26 | 0.07584 |
| CX3CL1 | Peprotech | 300-31 | 0.07273 |
| CXCL1 | Peprotech | 300-11 | 0.06253 |
| CXCL3 | Peprotech | 300-40 | 0.04122 |
| CCL7 | Peprotech | 300-17 | 0.03498 |
| CCL28 | Peprotech | 300-57 | 0.03433 |
| CXCL4 | Peprotech | 300-16 | 0.03391 |
| CXCL5 | Peprotech | 300-22 | 0.02478 |
| CXCL11 | Peprotech | 300-46 | 0.01914 |
| CCL23 | Biolegend | 587002 | 0.01151 |
| XCL1 | Peprotech | 300-20 | 0.01034 |
| CCL26 | Peprotech | 300-48 | 0.01013 |
| CXCL6 | Peprotech | 300-41 | 0.0101 |
| CCL16 | Peprotech | 300-44 | 0.00895 |
| CXCL13 | Peprotech | 300-47 | 0.00856 |
| CCL20 | Peprotech | 300-29A | 0.00822 |
| CCL22 | Peprotech | 300-36A | 0.00705 |
| CCL24 | Peprotech | 300-33 | 0.00689 |
| CCL17 | Peprotech | 300-30 | 0.00334 |
| CCL11 | Peprotech | 300-21 | 0.00316 |
| CXCL14 | Peprotech | 300-50 | 0.0018 |
| CCL15 | Novus | NBP2-35043 | 0.00149 |
| CCL27 | Peprotech | 300-54 | 7e-04 |
| CCL1 | Peprotech | 300-37 | 0.00042 |
